# Regulation and Compliance in Telemedicine: Viewpoint

**DOI:** 10.2196/53558

**Published:** 2025-01-23

**Authors:** Julia Ivanova, Mollie R Cummins, Triton Ong, Hiral Soni, Janelle Barrera, Hattie Wilczewski, Brandon Welch, Brian Bunnell

**Affiliations:** 1 Doxy.me Research Doxy.me, Inc Charleston, SC United States; 2 College of Nursing University of Utah Salt Lake City, UT United States; 3 Department of Psychiatry and Behavioral Neurosciences Morsani College of Medicine University of South Florida Tampa, FL United States; 4 Biomedical Informatics Center Medical University of South Carolina Charleston, SC United States

**Keywords:** telemedicine, telehealth, policy, COVID-19, PHE, rules and regulations, compliance, privacy and security, regulation, rule, public health, US, United States, implementation, regulatory, professional, organizational, ethical, concern, privacy, security, government literature, law, health care, patient

## Abstract

The US COVID-19 Public Health Emergency ended on May 11, 2023. Lawmakers and regulators extended some flexibilities while they deliberate effective long-term telemedicine policy. Here, we discuss critical challenges in telemedicine compliance and regulation grounded in scholarly literature and current events. We specifically consider obstacles and progress toward solutions in telemedicine law and regulation regarding privacy and security issues, care across state borders, and prescribing over telemedicine in the United States. We conclude that simplified policies are needed to keep telemedicine accessible to providers and patients and that current privacy and security measures need refinement to protect patients appropriately.

## Introduction

### Background

Smartphones and the internet have had a transformative impact on telemedicine over the decades, but the tipping point for telemedicine adoption was the COVID-19 pandemic [1]. During the pandemic, numerous health professionals adopted telemedicine to provide care while decreasing exposure to the virus [2]. In the US, legal and regulatory flexibilities enabled the shift of health care services to telemedicine. The Coronavirus Aid, Relief, and Economic Security Act and the Public Health Emergency (PHE) protocols of 2020 implemented most pandemic-era policy flexibilities in the United States, including (1) relaxation of HIPAA (Health Insurance Portability and Accountability Act) compliance requirements, (2) permission to prescribe certain controlled substances via telemedicine, (3) reimbursement for telemedicine equivalent to that of in-person visits, (4) promotion of telemedicine expansion for Medicare, (5) allowances for providers to offer care across state borders regardless of licensure, and (6) new protocols and *ICD-10* (*International Statistical Classification of Diseases, Tenth Revision*) codes for telemedicine visits [3,4]. Lawmakers made these changes to increase access to health care nationwide during the pandemic and based these flexibilities on previously considered legislative, regulatory, and policy updates [5].

The expiration of the PHE in May 2023 created a dilemma for lawmakers, who were unprepared to make decisions about long-term telemedicine policy. To avoid negative impacts and allow more time for decision-making, they extended many telemedicine-related policy flexibilities through December 31, 2024 [6,7]. Now, regulatory leaders are tasked with assessing current evidence (eg, telemedicine research, stakeholder needs and experiences, security concerns, cases of crime, and fraud) to make informed decisions about permanent federal and state regulations and rules related to telemedicine [8].

At this critical juncture, it is essential that we consider outcomes, experiences, perspectives, and other evidence resulting from pandemic-era telemedicine policy. By doing so, we can identify critical challenges and potential solutions and ultimately make informed, long-term policy decisions. Here, we summarize high-priority, policy-related challenges, and proposed solutions, focusing on providers and patients. We argue for US federal and state policy that protects patients in the context of new technologies and supports providers’ inclusion of telemedicine in their health care services. We highlight how ambiguities and complexity of laws and regulations effectively obstruct providers and organizations from using telemedicine. This viewpoint is grounded in a literature review of US telemedicine laws and regulations between 2000 and 2022 using legal and scientific databases (ie, HeinOnline, LexisNexis, PubMed, and Google Scholar) and includes post-PHE literature and events to ensure the relevancy of the topics discussed.

## Security and Privacy

HIPAA—known for its privacy and security rules establishing national standards for protecting personal health information (PHI)—mandates providers use telemedicine platforms that provide privacy and security by not storing or having sufficient ways to prevent breaches of PHI [9]. The PHE flexibilities waived the need to use HIPAA-compliant telemedicine platforms. This flexibility eased the rapid, large-scale transition to telemedicine use during COVID-19 [9,10]. Unfortunately, HIPAA does not cover all facets of digital practices in health care delivery. For example, third-party digital apps for patients to complete therapies or exercises may not be required to comply with HIPAA, even if providers use it as an adjunct to care [11-13]. Providers may use a HIPAA-compliant platform, but patients may be unknowingly in a nonprivate space. Simply expanding the scope of HIPAA to include PHI shared outside of a direct patient-provider relationship may not be enough protection [13]. Even in 2018, legal commentary noted that updating the laws and regulations to protect PHI through more rigorous cybersecurity expectations, such as requiring encryption of transmissions and concrete language regarding security measures, is critical in ensuring the security and privacy of PHI [13]. HIPAA’s security rules should stipulate the use of feasible cybersecurity measures rather than the basic health care standards [13]. A health care practice could determine the best cybersecurity measures that would balance the importance of privacy and security of their patients while ensuring such measures are not a costly burden. To protect patients and providers, we must reconsider emphasizing the best instead of minimum necessary practices regarding privacy and security laws in this new age of digital health telemedicine and apps.

While providers have stated expectations that HIPAA amendments increase protections [14,15], the proposed changes focus on improving patient access to their PHI and easing administrative burdens [16]. These proposed changes would only apply to the current definition of covered entities, and therefore, will not address the challenges regarding third-party health apps. Though HIPAA is the federal standard for PHI protection, state laws can enhance protection above this minimum requirement.

At the state level, the issue of PHI protection has led to the enactment of Washington state’s My Health My Data Act (HB 1155), whereby the state protects PHI beyond the scope of HIPAA [17]. Specifically, this law prevents businesses from inferring health data about an individual based on purchases. It requires regulated entities or businesses to have information on this law within their privacy policies [17]. The My Health My Data Act reflects a general movement toward increased security of individual health information. As the first to pass, the law broadly applies to consumer health data and includes information that may be inferred to determine a person’s past, current, or future health [18]. Based on the discussions around this and similar proposed laws, we expect to see more proposed state and federal legislation with varying degrees of protection, greatly based on the ability of the government and institutions to enforce such protections [19-21]. With no current tangible proposal to amend HIPAA in this way, state legislatures are stepping up their security and protections of PHI: Nevada passed their consumer health law in 2023, though their definitions of health data and protections are more narrowly defined than those of Washington’s law [22].

At this time, planned HIPAA amendments focus on increasing patient access to their PHI and would not directly address concerns related to digital tools. Some states are attempting to remedy privacy and security issues identified with the increased use of telemedicine and new technologies. Though their initiative raises awareness and discussion of privacy and security related to telemedicine, the lack of streamlining may increase legal complexities. Until there is a concrete effort to streamline these issues, telemedicine providers must ensure compliance with new state laws and regulations, and patients must be mindful of how they share PHI.

## Prescribing Over Telemedicine

The PHE flexibilities waived the requirement to see a patient in person before prescribing controlled medication including schedule II-V drugs. Before the flexibilities, the Drug Enforcement Administration (DEA) required providers to obtain a waiver for each state where they prescribe medication. The DEA waived this step during the PHE [23]. Still, providers had to consider different state laws regulating their prescribing ability [24]. The DEA expects to promulgate its final rule incorporating provider and patient expectations since the PHE into its regulations in 2025. Due to this extended timeline, experts' expectations of a third extension of PHE flexibilities was confirmed with the DEA's clean extension of these flexibilities through December 31, 2025 [23,24].

Patient and provider experiences with these flexibilities have led to robust discourse, especially over the dangers—or lack thereof—of prescribing buprenorphine (schedule III drug) without in-person visits [25]. During the pandemic, providers reported that reduced steps in monitoring made them rely more on patients’ decision-making and confront the issue of trust in their patient-provider relationships [26]. However, the same providers noted the ability to see patients in their homes provided them with a new level of insight that they used in assessing their patients [26]. Similarly, a qualitative analysis of provider experiences in prescribing medications for opioid use disorder indicated both positive—including increased insight into patients’ daily lives—and negative experiences, noting issues with patients viewing telemedicine as a less professional encounter [27]. Providers additionally experienced stress due to their uncertainty regarding prescribing laws [27].

Research on prescribing over telemedicine considered patient outcomes, as well as provider experiences. A study examining Medicaid data during the beginning of the pandemic found that patients who began buprenorphine treatment over telemedicine had higher treatment retention, and there was no change in the odds of opioid-related nonfatal overdose [28]. A recent review concluded that most providers favored having the ability to prescribe buprenorphine over telemedicine with little to no issues [29]. While the evidence is not definitive, providers appear to favor having the option of prescribing buprenorphine over telemedicine. However, providers note certain contextual misgivings such as the potential for increased liability and lack of legal clarity [29]. Though evidence appears to show that telemedicine increases access and results in similar efficacy as in-person treatment using medications for opioid use disorder, the research is still ongoing [30,31].

Ideally, there will be a continuation of rigorous research to determine how prescribing buprenorphine—and other controlled substances—over telemedicine affects efficacy, access, health inequalities, and patient safety [31]. Unfortunately, with the DEA’s current timeline, new research is unlikely to inform the final rule. Instead, the DEA will make its decision based on current evidence, the 38,000 written comments providing feedback to its proposed rule, and the two days of public listening sessions that occurred in September 2023 [6]. The DEA’s final rule is the first step to simplifying the laws and regulations surrounding prescribing over telemedicine, mainly controlled substances. We expect to see moderately more expansive flexibilities than the initially proposed ruling to support the necessary accessibility of opioid use disorder treatment for individuals. Additionally, should the DEA remove the requirement for individual waivers to prescribe in each state, there would be a significant streamlining of procedures providers must follow to prescribe over telemedicine. Considering the DEA’s development of a federal ruling, eliminating the required multiple waivers would align with the premise of reducing federal compliance and regulatory complexities.

The DEA’s final rule also affects how states make rules and regulations. For example, in late 2023, the Georgia Medical Board planned to revert its prescribing policies to prepandemic times. After discussions with the community, it decided to extend PHE flexibilities on the state level through January 31, 2025. The board is waiting to see the DEA’s final ruling before determining its state policy on the subject [32]. As with many federal and state dynamics, states will examine national rules and regulations to determine their policies. Based on the DEA's final ruling, we expect to see further changes to laws and regulations at the state level.

## Expansion of Medicare, Coverage, and Reimbursement

A widely cited barrier to telemedicine use is the issue of payment, both for coverage and reimbursement. Parity laws relate to payment for telemedicine services and include coverage parity (insurers must pay for services) and payment parity (insurers must pay for services at an equal rate to in-person visits) [33]. With the PHE flexibilities, coverage parity ensured that telemedicine services were provided in all states and through Medicare [34]. Additionally, the flexibilities implemented payment parity for telemedicine services through Medicare so that providers were paid at the same rate as they would for an in-person visit [35]. During this time, most commercial insurers also provided payment parity [36].

Payment parity on the state level has changed during the COVID-19 era (Table 1) [35]. On a federal level, Medicare payment parity continues through the end of 2024; however, reimbursement after this extension will depend on Medicare’s telemedicine code categories [37].

**Table 1:** Number of states with pre-, peri-, and post-PHE^a^ payment parity laws.

		States, n
**Pre-PHE**		
	Medicaid	4
	All insurers	11
**PHE**		
	Medicaid	14
	All insurers	20
**Post-PHE**		
	Medicaid	6
	All insurers	21

^a^PHE: Public Health Emergency.

At this point, it is unknown whether Medicare will implement permanent changes on the federal level and whether certain states will permanently continue payment parity for telemedicine services. Differences in state and federal laws can impede health care providers’ ability to navigate payment, reducing patient health care access [37]. These laws burdened providers with understanding the changing legal landscape of telemedicine and determining whether it is fiscally feasible.

Overall, there is federal coverage parity through Medicare, and all 50 states and Washington, DC, offer coverage parity with some variation in language [34]. While there is uncertainty regarding payment parity continuance through Medicare, the physician fee schedule for 2024 posted for Medicare included payment parity and granularity in types of telemedicine visits [38]. On the state level, payment parity continues to evolve. States look to one another to determine what has worked effectively and what has not in choosing and implementing their parity laws. State and federal legal definitions of telemedicine visits may differ. Still, there is clear support for ensuring that telemedicine is sustained as a health care delivery option in the United States. We see the trend that state laws will eventually conform to those of Medicare, resulting in unified definitions of telemedicine and its reimbursement. Until this happens, providers may continue to view payment parity as a hindrance to implementing telemedicine in their workflow.

## Care Across State Borders

During the PHE, providers could deliver health care nationwide with any state license. Unlike the DEA’s extension of the flexibility—allowing a provider to have a single waiver to prescribe controlled substances nationwide—lawmakers did not extend the PHE flexibility that allowed providers to care for patients across state lines without a license in each state. States have varied in their regulation of telemedicine across state borders [39]. For example, if a provider is licensed in New York and treats a patient who is in California for college or lives part-time in Florida, they can only see the patient within New York (even if the provider is out of state during the visit) [40]. In the same example, if the New York provider has a patient in New Jersey, that patient would have to drive into New York to be seen over telemedicine. Such requirements have created the telemedicine parking lot phenomenon wherein patients drive to the nearest location within the provider’s state and park to be seen via telemedicine to comply with state laws and licensure [41].

Regulatory changes allowing providers to see patients outside their states may increase patient safety and continuity of care [42,43]. A policy initiative, supported by professional organizations, such as the American College of Physicians and the American Medical Association, would ease regulatory compliance and eliminate a significant barrier to telemedicine care delivery across US state borders [13,43-46]. Licensure compacts exist for health professionals outside physicians, that is, nurses, physical therapists, occupational therapists, speech-language pathologists, audiologists, psychologists, and emergency medical professionals, that enable practice across multiple states [47]. Unfortunately, not all compacts act in the same way: while the Interstate Medical Licensure Compact for physicians only helps to streamline the licensing process, the Nurse Licensure Compact allows nurses to hold one license and practice in all participating states [33]. The larger scope of the Nurse Licensure Compact overcomes the additional barriers of time, complexity, and money that providers face. Of course, with each licensure compact, there are rules and regulations at the state level that would require amendment including definitions and functions of noncompete agreements and standards of care [48-51]. Ultimately, licensure compacts help simplify and codify the laws and regulations surrounding practice across state lines. Lawmakers and regulators need to remedy the level of complexity in the state and federal legislation in the face of the patient’s needs and the providers’ abilities [52]. Since the PHE, more states have joined or are in the process of joining these licensure compacts. For example, the Interstate Medical Licensure Compact currently includes 39 states and Washington, DC, and the Nurse Licensure Compact consists of 41 states [40,53].

## Conclusions

With the end of the PHE, US Congress and regulators allowed certain telemedicine policy flexibilities to continue temporarily. At the same time, lawmakers and regulators work to align pre-PHE policies with current provider and patient needs relating to telemedicine. Over 100 pieces of proposed federal legislation currently address aspects of telemedicine, with more proposals from state lawmakers [39]. This viewpoint reviewed how COVID-19–era PHE flexibilities paved the way for telemedicine adoption, summarized the policy challenges that arose, and described how policy leaders are attempting to resolve the challenges.

In the coming months and years, we anticipate changes to privacy and security laws, potential changes to HIPAA, and increasing consideration of how privacy laws function concerning emergent digital health technologies. After the DEA's final ruling regarding prescribing over telemedicine, expected in 2025, we anticipate further state-level changes in laws and regulations to better align with the federal standard. We anticipate that current regulatory trends supporting telemedicine coverage and payment parity will continue and that more states will adopt interstate licensure compacts for their health care professionals. Overall, our assessment of these critical topics reveals that while telemedicine is now an unmistakable part of health care, there is a need to codify the laws and regulations on a federal and state level. Such a move would ensure patient privacy, security, and quality of care and ensure provider support through definitive, clear pathways to comply with telemedicine laws and regulations.

## Abbreviations

DEA: Drug Enforcement Administration
HIPAA: Health Insurance Portability and Accountability Act
ICD-10: International Statistical Classification of Diseases, Tenth Revision
PHE: Public Health Emergency
PHI: personal health information
